# Metformin and Its Benefits for Various Diseases

**DOI:** 10.3389/fendo.2020.00191

**Published:** 2020-04-16

**Authors:** Ziquan Lv, Yajie Guo

**Affiliations:** ^1^The Eighth Affiliated Hospital, Sun Yat-Sen University, Shenzhen, China; ^2^Department of Molecular Epidemiology, Shenzhen Center for Disease Control and Prevention, Shenzhen, China

**Keywords:** metformin, benefits, diseases, mitochondrial respiratory chain complex I, AMPK

## Abstract

Metformin is a widely used biguanide drug due to its safety and low cost. It has been used for over 60 years to treat type 2 diabetes at the early stages because of its outstanding ability to decrease plasma glucose levels. Over time, different uses of metformin were discovered, and the benefits of metformin for various diseases and even aging were verified. These diseases include cancers (e.g., breast cancer, endometrial cancer, bone cancer, colorectal cancer, and melanoma), obesity, liver diseases, cardiovascular disease, and renal diseases. Metformin exerts different effects through different signaling pathways. However, the underlying mechanisms of these different benefits remain to be elucidated. The aim of this review is to provide a brief summary of the benefits of metformin and to discuss the possible underlying mechanisms.

## Introduction

Metformin, a derivative of biguanide, is one of the most commonly used drugs to treat type 2 diabetes (T2D), and it has been used for nearly one century (1). Guanidine was found to have anti-diabetic properties in animals in 1918, but unfortunately, it was toxic in clinical trials (2, 3). This prompted scientists to find safer substitutions. In the 1920s, metformin (1,1-dimethyl biguanide hydrochloride) was synthesized. Since then, metformin became the first choice to treat T2D due to its remarkable ability to decrease plasma glucose levels (3–5). In recent years, many additional unexpected but effective roles of metformin were found. Studies showed that metformin exerts a strong effect on numerous cancers (6, 7), cardiovascular disease (CVD) (8), liver diseases (9), obesity (10), neurodegenerative diseases (11), and renal diseases (12). Sole medication or combination therapy with other drugs has shown to be effective to treat different diseases.

Metformin inhibits mitochondrial complex I (13, 14), which leads to AMPK (adenosine 5′- monophosphate—activated protein kinase) activation (15). Mitochondrial complex I is vital to electron transport. As a result, the production of ATP (adenosine triphosphate) decreases and the intracellular concentration of ADP (adenosine diphosphate) increases. Consequently, the cellular levels of AMP (adenosine monophpsphate) increases, finally activating AMPK (14, 16). Moreover, a recent study showed that metformin could activate AMPK via the lysosomal pathway, i.e., the AXIN/LKB1-v-ATPase-Regulator pathway (17). AMPK is a key regulator of numerous metabolic pathways, including glucose metabolism, lipid metabolism, and energy homeostasis (18, 19). Besides, metformin plays important roles by inhibiting insulin and IGF receptor signaling, resulting in changes in metabolic homeostasis (20). Recently, Using *hd*PCA (“homomer dynamics” protein-fragment complementation assays), a strategy that is able to map out gene functions and target pathways of drugs, it was found that the levels of proteins which were responsible for a broad set of cellular processes, including energy metabolism, aging, and cancer, were changed by metformin (21). The underlying mechanisms of metformin in the regulation of diseases, however, are still not fully understood. Here, we summarize the functions of metformin and discuss the underlying mechanisms from various perspectives, which may help to provide insight for future investigations.

## Metformin and Diabetes

Numerous studies and clinical trials have demonstrated that metformin monotherapy or combination therapy with other glucose-lowering drugs is effective in treating T2D. A report from 1995 illustrated that metformin is able to lower plasma glucose levels, and in the decades that followed, new roles of metformin in diabetes have been discovered. In the 1995 study, by Defronzo et al., 289 diabetes patients were treated with metformin or placebo. After 29 weeks, the metformin group showed lower mean fasting plasma glucose and HbA_1c_ levels (22). In a 1997 study by Garber, 451 diabetic individuals were given different dosages of metformin (ranging from 500 mg to 2,000 mg daily). After 14 weeks, it was found that metformin's efficacy is dose-dependent (23). In 2006, a 5-year randomized and double blind clinical trial in which metformin was compared with glibenclamide and rosiglitazone, other anti-diabetic drugs, was published. The results showed that the fasting plasma glucose levels were decreased the least by rosiglitazone and the most by glibenclamide, with metformin showing intermediate effects (24).

In some cases, metformin is used in combination with other anti-diabetic drugs or reagents. For example, in a 29-week study of 632 individuals, the combination of metformin and glibenclamide showed better glucose control than metformin alone (22). Glimepiride showed similar results in a clinical trial with 372 individuals (25). Another study showed that metformin and troglitazone lead to a stronger reduction in fasting plasma glucose and postprandial glucose levels after 3 months of treatment than treatment with metformin alone (26). Moreover, studies demonstrated that combination therapy of metformin with DPP4 inhibitors, SGLT2 inhibitors, or GLP1 receptor agonists also showed effective glucose control, without an additional risk of hypoglycaemia (27, 28). Combination of metformin and insulin is another way to treat diabetes. In a trial with 96 patients, this combination exhibited better control of glucose levels and weight gain than treatment with metformin alone (29). In another study, with 390 patients, the combination with insulin also exhibited better glucose control than treatment with metformin alone (30).

Moreover, metformin improves insulin sensitivity and decreases fasting insulin levels in cognitive impairment patients with abnormal glucose metabolism (31). Metformin is a rational treatment choice for pregnant women with T2D, gestational diabetes (GDM), and polycystic ovarian syndrome (PCOS). Metformin was shown to have a stronger reducing effect on the body weight of PCOS patients than rosiglitazone. On the basis of *in vitro* and *in vivo* studies, including animal studies and clinical trials, the use of metformin in pregnancy is becoming increasing common globally (32). Nevertheless, the safety is controversial. Studies showed that children exposed to metformin may have a higher prevalence of obesity, BMI, abdominal fat volume, or blood pressure (33, 34). Other research suggested that patients taking metformin for more than 10 years had an increased risk of beta cell failure and insulin resistance (35). Although long follow-up studies may be required to explore the possible effects of metformin on human cells and tissues, metformin is undoubtedly the preferred treatment option for diabetes patients.

Metformin exerts its anti-hyperglycemic effects mostly by suppressing hepatic glucose production through AMPK-dependent (36, 37) or -independent pathways (38, 39). On the one hand, metformin inhibits gluconeogenesis through AMPK-dependent activation of SHP (small heterodimer partner) and inhibition of phosphorylation of CBP (CREB binding protein) (40), thus suppressing the expression of gluconeogenic genes, such as *G6Pase* (glucose 6 phosphatase), *PEPCK* (phosphoenolpyruvate carboxykinase), and *PC* (pyruvate carboxylase) (41). Moreover, activation of AMPK leads to the inhibition of mTORC1 (mammalian target of rapamycin complex I), which also results in the suppression of gluconeogenesis (42). On the other hand, metformin inhibits hepatic glucose production in an AMPK-independent manner. Studies showed that metformin attenuates the ability of glucagon (43) or inhibits mitochondrial GPD (glycerol-3-phosphate dehydrogenase), subsequently leading to an impairment of lactate utilization for gluconeogenesis (39). Recently, a study also demonstrated that metformin directly targets FBP1 (fructose-1,6-bisphosphatase-1), the rate controlling enzyme in gluconeogenesis, inhibiting hepatic glucose production (44). Other studies suggested that metformin could also enhance GLUT1 (glucose transporter 1) mediated glucose transport into hepatocytes through activating IRS2 (insulin receptor substrate two), decreasing plasma glucose levels (45).

Besides decreasing liver glucose production, metformin also decreases glucose levels through increasing (i) GLUT4(glucose transporter 4) mediated glucose uptake in skeletal muscles (46) and (ii) absorption of glucose in the intestines (47). Metformin also stimulates GLP-1 (glucagon-like-peptide-1) release, thereby enhancing insulin secretion and lowering plasma glucose levels. Moreover, recent studies suggested that gut microbiota may be a target site of metformin. An increasing number of studies have showed dysbiosis of the gut microbiota in T2D patients (48, 49). In a randomized, double blind study, scientists found that metformin affects the composition and function of the gut microbiota (50), providing new insight in the mechanism underlying metformin's anti-diabetic effects. After a short-time administration of metformin, the *Bacteroides fragilis* count in the gut decreased, which resulted in an increase in GUDCA (glycoursodexoycholic acid) levels. The elevation of GUDCA levels suppresses intestinal FXR (farnesoid X receptor), which improves glucose tolerance (51).

## Metformin and Cancer

Accumulating evidence indicates that metformin inhibits growth, survival, and metastasis of different types of tumor cells, including those from breast, liver, bone, pancreas, endometrial, colorectal, kidney, and lung cancers (52). Metformin's anti-cancer properties depend on its direct and indirect regulation of cells' metabolism. The direct effects are mediated by AMPK-dependent and -independent pathways. (i) Metformin activates AMPK, which leads to the inhibition of mTOR signaling, and as a result, protein synthesis is disturbed, and cell growth and proliferation is suppressed (53). For example, crosstalk between G protein-coupled receptors (GPCRs) (54) and insulin receptor signaling systems may be inhibited by metformin: possibly contributing to the inhibition of pancreatic cancer proliferation (55). P53 is considered as a critical tumor suppressor gene in human cancers (56). Research showed that p53 is involved in the anti-cancer effects of metformin (57). Metformin activates AMPK and then induces p53 phosphorylation to prevent cell invasion and metastasis (57). (ii) Metformin also inhibits mTORC1, a key regulator of cell growth that can integrate intracellular and extracellular stimuli (58), in an AMPK-independent manner (59). Additionally, metformin suppresses mitochondrial complex I, thereby preventing the generation of reactive oxygen species (ROS) and further decreasing DNA damage, suppressing cancer development (60). Previous studies also suggested that metformin can suppress cancer development by activating autophagy and apoptosis through an AMPK-independent pathway.

Considering the indirect beneficial effects of metformin in cancer, studies indicated that metformin could regulate angiogenesis, fibroblasts, tumor-associated macrophages, and immunosuppression, changing the tumor microenvironment (61). As an anti-diabetic drug, metformin decreases plasma glucose levels, thereby inhibiting cancer cell proliferation and survival (62). Other studies reported that metformin could activate the immune response against cancer cells (16) or decrease NF-κB (nuclear factor-κB) activity, which results in a reduction in the secretion of pro-inflammatory cytokines (63). In addition, microRNA has been suggested to mediate one of the anti-cancer actions of metformin. Studies showed that metformin could induce DICER expression *in vitro* and *in vivo*, a crucial enzyme in the regulation of microRNA biogenesis (64). Recently, a study found that metformin combined with fasting-induced hypoglycemia synergistically impairs tumor metabolic plasticity and growth via the PP2A/GSK3β/MCL-1 axis (65). It has been suggested that tumor cells alternate between glycolysis and oxidative phosphorylation (OXPHOS) to adapt to metabolic challenges. Dietary limitation through intermittent fasting (IF) is an emerging approach to inhibit tumor development (66), while metformin is an OXPHOS inhibitor. It has been found that combination of metformin and intermittent fasting showed the strongest reduction in tumor growth without causing any weight loss or toxicity. This suggests more potential strategies to treat tumors with metformin may be developed in the future.

### Breast Cancer

Breast cancer (BC) is one of the most common malignancies occurring in females. It is driven by a multitude of cellular pathways and its incidence increases with age (67). Cellular glucose metabolism is linked tightly with the proliferation and development of breast cancer. Several studies suggested that metformin reduces the incidence of breast cancer in T2D patients (68). Cancer cells show enhanced glucose uptake and metabolism and prefer glycolysis over OXPHOS, which is called the “Warburg effect.” The noted specialty of metformin is to decrease glucose levels, thereby limiting the availability of energy for cancer cells. Metformin was also shown to decrease FAS expression, an essential component of the fatty acid synthesis pathway, thus affecting the survival of cancer cells.

Triple negative breast cancer (TNBC) is a kind of breast cancer that is difficult to cure, due to the lack of approved targeted therapies and effective chemotherapy with low toxicity (69). BACH1 (BTB and CNC homology 1) was reported to be the main regulator of glycolysis and OXPHOS in TNBC, and is therefore related to the Warburg effect. A previous study has showed that heme could suppress the expression of BACH1 and is helpful in the treatment of TNBC (70). Recently, a study indicated that combination therapy of heme and metformin significantly inhibits tumor growth and strongly suppresses TNBC (71). These findings provide us with new insight in the use of metformin combined with other drugs to treat tumors.

### Blood Cancer

In the progression and treatment of multiple myeloma (MM), AKT signaling occupies an important place. In MM, AKT expression is always high, even in the advanced stages (72). Studies showed that metformin inhibits AKT/mTOR signaling, thereby impairing MM cell proliferation. Furthermore, metformin could also inhibit GRP78 (glucose regulatory protein 78) to further impair autophagosome formation and increase apoptosis, strengthening the anti-myeloma effects of brotezomib (73).

Leukemia comprises 2.8% of all cancers and 3.4% of cancer-related deaths worldwide. The aberrant activation of the PI3K/AKT/mTOR pathway is one of the most common biochemical features of leukemia (74). Metformin inhibits AKT/mTOR signaling, and might therefore be an effective approach to treat leukemia. Metformin has a beneficial role in human lymphoma by inhibiting mTOR signaling without the involvement of AKT, and the suppression of mTOR subsequently leads to the suppression of growth of B cells and T cells (75).

### Colorectal Cancer

Colorectal cancer (CRC) is also one of the most common cancers in the world, with an increasing incidence in low and middle income countries. Recently, numerous studies, including fundamental research, clinical trials, and epidemiological studies, showed that metformin might be a candidate chemoprevention drug to decrease the risk of CRC development. In 2004, a report had demonstrated the relationship between metformin and CRC (76), and in the following years, the beneficial effects of metformin on the regulation of CRC development were reported in several studies (77). Metformin may exert its pharmacodynamic effects through the gut-brain-liver axis, but these mechanisms require further exploration. In the intestine, metformin increases glucose uptake and lactate concentrations. Metformin administration increases the bile acid pool in the intestine, which may affect GLP-1 secretion and cholesterol levels. In addition, metformin changes the microbiome, affecting the regulation of metabolism, such as glucose homeostasis, lipid metabolism, and energy metabolism (78). These changes contribute to the inhibition of the development and progress of CRC.

### Endometrial Cancer

Endometrial cancer is the fifth most common malignancy in women with the incidence rising in both developed and developing countries (79). Disordered metabolism caused by metabolic syndrome like obesity and hyperglycemia is related to the development of endometrial cancer. Metformin is an effective anti-diabetic drug, studies have demonstrated the beneficial effect of metformin on endometrial cancer development. Studies showed metformin administration improves survival rate in diabetic patients with endometrial cancer (80). The mechanisms involved in the effect of metformin in treating endometrial cancer are mainly mitochondrial OXPHOS suppression and AMPK activation, which subsequently inhibiting a variety of metabolic pathways, including STAT3, ZEB-1, ACC, mTOR, and IGF-1. These leads to protein synthesis and fatty acid synthesis decreased, apoptosis and autophagy increased, cell proliferation and cell cycle progression decreased, which all have a contribution to the suppression of endometrial cancer.

### Melanoma

Melanoma is the most aggressive skin cancer and is responsible for almost 80% of the skin cancer-related deaths. Due to its strong invasive ability, melanoma often metastasizes to the lymph nodes, liver, lungs, and even the central nervous system (81). Because of its strong resistance to therapies and the ability to escape from the immune response, melanoma is a difficult public health problem. Currently, two antibodies for the treatment of melanoma are available, i.e., ipilimumab (anti-CTLA-4) and nivolumab (anti-PD-1) (82). However, 55–60% patients do not respond to these treatments, and new treatment strategies are urgently required. Metformin can induce cell cycle arrest in the G0–G1 phase in melanoma cells. Another study indicated that metformin can attenuate melanoma growth and metastasis through inhibiting the expression of TRB3 (tribbles pseudokinase 3) in non-diabetic and diabetic mouse models (83). Because of the activation effect of AMPK, metformin could influence melanoma cell death and proliferation and the tumor microenvironment. It will be interesting to investigate the effects of combination treatment of metformin with current therapies or other drugs to treat melanoma.

### Bone Cancers

Compared with cancers initiating in bone tissue itself, invasion of metastatic cancers, especially breast, lung, and prostate cancers, into bones is more common (84). All types of bone cancers influence the osteolytic process, and osteoblastic metastases occur through osteoclast activation or stimulant factors which are responsible for osteoblastic proliferation, differentiation, and formation (85). RANKL (receptor activator of nuclear factor kappa-B ligand) is important in the suppression of osteoclast proliferation and differentiation, which is inhibited by AMPK upon metformin treatment. Moreover, metformin suppresses bone cancer cell proliferation, migration, and invasion via the AMPK/mTOR/S6 or the MMP2/MMP9 signaling pathway (86).

## Metformin and Obesity

The incidence of obesity has rapidly increased in recent years due to changes in lifestyle. Obesity is a multi-factor chronic disease accompanied with other related metabolic syndromes, such as diabetes, fatty liver diseases, and CVDs. Obesity is caused by an imbalance between energy intake and expenditure (87). Accumulating evidence suggests that metformin may be a potential therapy for obesity and its related metabolic dysfunctions. In non-diabetic individuals, metformin was shown to exert weak but beneficial effects on weight loss. In mice, metformin treatment for 14 weeks significantly prevented high-fat diet induced obesity and the associated inflammatory response through increasing the expression of FGF21 (fibroblast growth factor 21), a key metabolic hormone that improves lipolysis in white adipose tissue to prevent fat accumulation (88). Moreover, metformin may prevent obesity in mice by increasing metabolic activity of brown adipose tissue (BAT), a tissue with abundant mitochondria. Through the action of UCP1 (uncoupling protein 1), BAT is able to dissipate chemically bound energy as heat, a process known as thermogenesis. By PET/CT imaging, it was found that metformin was mainly taken up by BAT *in vivo* through the increased expression of OCT (organic cation transporter) (10). It was shown that metformin exerts its anti-obesity effects through increasing mitochondrial biogenesis, decreasing fatty acid uptake, and stimulating thermogenesis (89). Moreover, a study indicated that in rats, metformin modulates gut microbiota and prevents high-fat diet induced obesity, which increased the abundance of short-chain fatty acid producing bacteria and decreased microbial diversity (90). The ability to increase glucose metabolism may also contribute to the attenuation of obesity.

## Metformin and Liver Diseases

The liver, which plays a critical role in the physiology of the whole body, especially glucose homeostasis and lipid metabolism, is the main target organ of metformin. Liver dysfunction may lead to many diseases, such as diabetes, non-alcoholic fatty liver disease (NAFLD), cirrhosis, non-alcoholic hepatitis (NASH), and hepatocellular carcinoma (HCC). Studies showed that metformin is safe in patients with cirrhosis and decreased the risk of death by 57%. In diabetic patients, metformin caused a 50% reduction in HCC incidence and improved survival mainly by influencing cell growth and angiogenesis through the PI3K/AKT/mTOR signaling pathway (91). However, the benefits of metformin in treating NAFLD and hepatitis are still controversial. In animal trials, it was found that metformin prevents the development of high-fat diet induced fatty liver disease in ob/ob mice, which displayed decreased liver triglyceride content (92). In humans, metformin was also found to reduce the incidence of fatty liver diseases and to cause a histological response (93). However, other studies showed that metformin failed to improve liver histology, hepatic steatosis, and inflammation (94). Fewer than 10 cases of metformin hepatotoxicity have been reported, which may be explained by the concomitant intake of other potentially hepatotoxic drugs (95).

The main contributor to NAFLD is the disorder of hepatic *de novo* lipogenesis, a process relevant to several transcription factors, including SREBP (sterol regulatory element-binding protein), ChREBP (carbohydrate response element-binding protein), and LXR (liver X receptor). These factors affect the expression of key enzymes of lipogenesis, such as FAS (fatty acid synthase) and SCD1 (stearoyl CoA desaturase 1) (96). Insulin resistance is also able to induce NAFLD by the famous “two-hit” theory. Metformin treatment induces AMPK activation, leading to a reduction in ACC (acetyl-CoA carboxylase) or SREBP1c inhibition, which in turn reduces fatty acid oxidation and suppresses fatty acid synthesis. Meanwhile, metformin modulates the synthesis of adipokines like TNF-alpha and IL-6, which increase fatty acid oxidation and decrease *de novo* lipogenesis (9). The mTOR pathway may play an essential role in these effects, specifically in HCC. It was also suggested that metformin could induce FGF21 expression, consequently preventing high-fat diet induced fatty liver disease in mice (88).

## Metformin and Cardiovascular Diseases

CVD is one of the main causes to death and disability in the world. The causes for CVD are versatile and include smoking, diabetes, obesity, hyperlipemia, and hepertension. Diabetes, both type 1 diabetes and T2D, is often found comorbid with CVD (97). Hyperglycemia induces oxidative stress, resulting in lipoprotein dysfunction and endothelial dysfunction, increasing the risk of CVD. Metformin, a common anti-hyperglycemic drug, was shown to decrease the incidence of CVD in diabetes patients. Through activating AMPK, metformin inhibits alpha-dicarbonyl-mediated modification of apolipoprotein residues, consequently ameliorating high-density lipoprotein (HDL) dysfunction and reducing low-density lipoprotein (LDL) modifications. Reductions in HDL dysfunction improve cholesterol transport and diminish the cardiovascular risk. Moreover, metformin improves endothelial oxidative stress levels and attenuates hyperglycemia-induced inflammation, decreasing the occurrence of CVD (98).

T2D is also associated with a higher incidence of heart failure. Reports indicated that patients with diabetes account for almost one-third of heart failure cases (99). It has been shown that metformin improves the myocardial energy status through ameliorating cellular lipid and glucose metabolism via AMPK (100). Recently, a randomized controlled trial in patients with coronary artery disease without diabetes demonstrated that metformin significantly reduces left ventricular hypertrophy (LVH), one of the most powerful prognostic factors in coronary artery disease. This study found that metformin largely reduced left ventricular mass indexed to height, left ventricular mass, body weight, and oxidative stress (101). Several studies have reported the benefits of metformin in CVDs and heart failure in patients with or without diabetes, and it will be interesting to explore more possible applications of metformin in the future.

## Metformin and Aging

Aging is considered as a fact of life that is unavoidable and is modulated by genetic and dietary factors. People's quality of life gradually worsens, eventually losing self-care ability and becoming hospitalized. From ancient to modern times, people have continuously searched for different kinds of drugs to increase their lifespan or their health span (102). The declining ability to regenerate damaged tissue and the deterioration in homeostatic processes are considered as biological features of aging (103). Aging increases the probability of many health outcomes, including diabetes, CVDs, coronary artery disease, cancer, depression, osteoporosis, and especially neurodegenerative diseases, such as Alzheimer's disease (AD) and Parkinson's disease (PD). Usually, the primary causes for aging are DNA damage and autophagy. Aging is a result of DNA damage, which can be induced by ROS, alkylation, hydrolysis, chemicals, and ultraviolet and other radiation (104). Genetic and environmental factors involved in the regulation of autophagy are also critical factors in the aging process (105). The clinical use of metformin in aging has just started. In *Caenorhabditis elegans*, metformin was shown to extend the lifespan, but no obvious effects on aging were observed in humans. However, metformin improves aging-related diseases, such as diabetes, CVD, and cognitive disorders, leading to an extended lifespan in these patients. In T2D patients, metformin decreased the risk of diabetes-related death by 42% (106). Another clinical trial demonstrated that metformin treatment for about 24 weeks improved cognitive performance and reduced depressive symptoms (107).

The mechanisms by which metformin affects the aging process are partly dependent on the regulation of glucose metabolism. By inhibiting mitochondrial complex I, metformin reduces endogenous production of ROS and subsequently decreases DNA damage (60). By activating AMPK, metformin is able to inhibit NF-κB signaling and attenuate cell inflammation (108). Metformin also leads to decreased insulin levels, and suppresses IGF-1 signaling and mTOR signaling, these all together resulting in suppression of inflammation and autophagy that is beneficial to the aging process (109). Besides, metformin was shown to have a function in the regulation of the microbiome, which may be another way to affect aging (110). In addition, metformin reduces neuronal injury and improves oxygen/glucose deprivation, thereby improving neuronal survival and neuroprotective functions (108). Due to its protective roles, metformin could be a good choice for pharmacological intervention against aging and aging-related diseases in individuals with or without diabetes.

## Metformin and Renal Diseases

Acute kidney injury (AKI) and chronic kidney disease (CKD) are two major renal diseases that are defined as a rapid loss of renal function that occurs in a few hours or days and a progressive loss of renal function that occurs in a few months or years, respectively (111). Effective treatments for these diseases are still lacking. Diabetes is considered as an important cause of renal diseases, and metformin is an interesting candidate to treat renal diseases, although its use was restricted previously (112). Studies showed that daily oral administration of metformin could ameliorate kidney fibrosis and normalize kidney structure and function. These effects may be mediated by the AMPK signaling pathway, which can regulate cell growth and energy utilization. Another study found that in a CKD mouse model, metformin could suppress kidney injury and improve kidney function, through AMPK-mediated ACC signaling (113). In humans, metformin is also beneficial to kidney diseases. Except for the effects in patients with diabetes-induced kidney injury, clinical trials suggested that continuous metformin administration improved renal function and survival in patients with AKI and CKD (114). It is worth to note that appropriate dosage of metformin is very important in the treatment for renal diseases. The mechanisms underlying these kidney protective roles of metformin may be related to the regulation of glucose utilization, the decrease in cell inflammation, and oxidative stress.

## Conclusions

**Figure 1:**
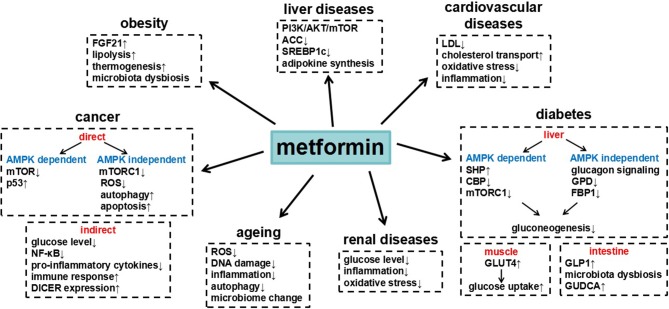
Summary of metformin in different diseases and the underlying major mechanisms.

Metformin is a widely used clinical drug with numerous benefits (Table 1), which through different signaling pathways (Figure 1). The most remarkable feature of metformin is anti-hyperglycemia. Cellular and animal studies have found that metformin inhibited the expression of gluconeogenic genes in AMPK dependent pathway or independent pathway, to suppress hepatic glucose production. Besides, metformin decreases glucose levels through impairing lactate utilization for gluconeogenesis, enhancing glucose transport and uptake, or changing gut microbiota. Accumulated clinical trials have evaluated that metformin had benefits on different cancers. Metformin prevents growth, survival, and metastasis of tumor cells and also changes the tumor microenvironment to suppress cancer development. The underlying molecular mechanisms include the inhibition of mTOR signaling, the activation of p53, autophagy and apoptosis, decreased generation of ROS, DNA damage and inflammatory response. Moreover, metformin was shown to have beneficial effects on liver diseases, obesity, cardiovascular diseases, age-related diseases, and renal diseases, thus finally decreasing death risk. These actions of metformin were related to AMPK activation or mitochondrial complex I inhibition, subsequently affecting cell metabolism. All these knowledge help us to understand the action of metformin on diseases, which may provide new potential therapeutic targets. The functions of metformin, however, are complicated and need to be explored further. Given its known safety and long-term use in humans, metformin is becoming a promising treatment option for many diseases.

**Table 1:** Beneficial effects of metformin on diseases.

**Disease**	**Subjects**	**Characteristics**	**Effects**	**References**
T2D	Human	289 patients receiving metformin (850 mg daily to 850 mg thrice daily) for 29 weeks	Fasting plasma glucose and HbA_1c_ levels decreased	(22)
	Human	451 patients receiving metformin (500–2,000 mg daily) for 14 weeks	Fasting plasma glucose levels decreased in a dose-dependent manner	(23)
	Human	5-year randomized and double blind clinical trial	Fasting plasma glucose levels decreased	(24)
	Human	632 individuals receiving metformin and glibenclamide for 29 weeks	Better glucose control than metformin monotherapy	(22)
	Human	372 individuals receiving metformin and glimepriride	Better glucose control than metformin monotherapy	(25)
	Human	Metformin and troglitazone treatment for 3 months	Lower fasting plasma glucose levels than metformin monotherapy	(26)
	Human	Combination of metformin with DPP4 inhibitors, SGLT2 inhibitors, GLP1 receptor agonists	Better glucose control than metformin monotherapy	(27, 28)
	Human	96 or 390 patients receiving metformin and insulin	Better glucose and weight gain control than metformin therapy	(29, 30)
	Human	Cognitive impairment patients with abnormal glucose metabolism receiving metformin	Insulin sensitivity improved, fasting insulin levels decreased	(31)
Breast cancer	Human	T2D patients receiving metformin	The incidence of breast cancer in T2D reduced	(68)
	Human tumor cells and mice	Combination therapy of heme and metformin	Tumor growth inhibited	(71)
Blood cancer	Cell lines and human	Stem cells or patients, metformin alone or combination with other agents	Cells growth and proliferation impaired	(73, 75)
Colorectal cancer	Cell lines and human	Cell lines or patients receiving metformin	The development and progress of CRC inhibited	(77, 78)
Endometrial cancer	Human	Diabetic patients with endometrial cancer receiving metformin	Survival rate of these patients increased	(80)
Melanoma	Human cells and mice	Cells or mice treated with metformin	Cell growth and metastasis inhibited	(83)
Bone cancer	Human and rat	Patients or rats receiving metformin	Cell growth and proliferation suppressed	(86)
Obesity	Human and mice	Metformin treatment in obese human and mice	Weight loss in human; improved lipolysis and thermogenesis in mice	(89, 90)
HCC	Human	Diabetic patients treated with metformin	The risk of death increased	(91)
NAFLD	Human and mice	Human or mice with fatty liver diseases treated with metformin	Liver triglyceride content decreased	(92, 93)
CVD	Human	Patients with or without diabetes receiving metformin	The occurrence of CVD decreased	(98, 101)
Aging	Human	Cognitive impairment patients treated with metformin	Aging related diseases improved and the risk of death decreased	(107–109)
AKI and CKD	Human	Patients receiving oral administration of metformin	Kidney structure and function improved	(113, 114)

## Author Contributions

ZL and YG researched data, wrote, reviewed, and edited the manuscript. YG had full access to all the data in the study and takes responsibility for the manuscript.

### Conflict of Interest

The authors declare that the research was conducted in the absence of any commercial or financial relationships that could be construed as a potential conflict of interest.

## Abbreviations

T2D: type 2 diabetes
CVD: cardiovascular disease
AMPK: adenosine 5'- monophosphate - activated protein kinase
ATP: adenosine triphosphate
ADP: adenosine diphosphate
AMP: adenosine monophpsphate
GDM: gestational diabetes
PCOS: polycystic ovarian syndrome
SHP: small heterodimer partner
CBP: CREB binding protein
G6Pase: glucose 6 phosphatase
PEPCK: phosphoenolpyruvate carboxykinase
PC: pyruvate carboxylase
mTORC1: mammalian target of rapamycin complex I
GPD: glycerol-3-phosphate dehydrogenase
FBP1, fructose-1: 6-bisphosphatase-1
GLUT1: glucose transporter 1
IRS2: insulin receptor substrate two
GLUT4: glucose transporter 4
GLP-1: glucagon-like-peptide-1
GUDCA: glycoursodexoycholic acid
FXR: farnesoid X receptor
ROS: reactive oxygen species
NF-κB: nuclear factor-κB
OXPHOS: oxidative phosphorylation
IF: intermittent fasting
BC: Breast cancer
TNBC: Triple negative breast cance
BACH1: BTB and CNC homology 1
MM: multiple myeloma
GRP78: glucose regulatory protein 78
CRC: Colorctal cancer
TRB3: tribbles pseudokinase 3
RANKL: receptor activator of nuclear factor kappa-B ligand
FGF21: fibroblast growth factor 21
BAT: brown adipose tissue
UCP1: uncoupling protein 1
OCT: organic cation transporter
NAFLD: Non-alcoholic fatty liver disease
NASH: nonalcoholic hepatitis
HCC: hepatocellular carcinoma
SERBP: sterol regulatory element-binding protein
ChREBP: carbohydrate response element-binding protein
LXR: liver X receptor
FAS: fatty acid synthase
SCD1: stearoyl CoA desaturase 1
ACC: acetyl-CoA carboxylase
HDL: high-density lipoprotein
LDL: low-density lipoprotein
LVH: left ventricular hypertrophy
AD: Alzheimer's disease
PD: Parkinson's disease
AKI: Acute kidney injury
CKD: chronic kidney disease.
